# Optimising MDT Huddles: A QIP Approach to Improving Efficiency and Satisfaction in an Older Adults Psychiatric Ward

**DOI:** 10.1192/bjo.2024.356

**Published:** 2024-08-01

**Authors:** Adnan-Mustafiz Chowdhury, Tharun Zacharia

**Affiliations:** South London and Maudsley NHS Foundation Trust, London, United Kingdom

## Abstract

**Aims:**

At Chelsham House – an older adults, acute inpatient dementia mental health ward – morning handover meetings (‘huddles’) lacked structure and consistency, resulting in extended, inefficient patient handover discussions and unclear task allocation. These issues consumed valuable clinical time and impacted the continuity and effectiveness of care. Recognising these challenges, a need to revamp the huddle format emerged, prioritising clear communication, effective task distribution, and team cohesion to enhance patient safety and care efficiency.

This project aimed to improve the efficiency and effectiveness of morning huddles at Chelsham House by reducing their average duration by 10% and enhancing multidisciplinary team (MDT) staff satisfaction regarding patient handover dialogues, task distribution, and accountability within 2 weeks.

**Methods:**

The intervention streamlined the huddle format by assigning a rotating MDT chairperson and task allocator, setting a strict 2-min per patient discussion target. New segments, such as a ward safety check and focused discussions on risks and discharge barriers within patient updates, were added. A task allocation board was implemented in the meeting room for assigning tasks. Staff surveys and data on meeting duration were collected pre- and post-implementation.

**Results:**

The implementation led to a 16% reduction in huddle duration (from 64 to 54 minutes) and a 21% decrease in time spent per patient discussion (from 4.09 to 3.23 minutes). Staff surveys showed a significant increase in satisfaction regarding safety discussions (21%), task clarity (23%), and discharge planning efficiency (26%). The effectiveness of mental and physical health discussions was maintained, with a high average Likert score of 4.64 post-implementation, on a scale where 1 is ‘Strongly Disagree' and 5 is ‘Strongly Agree'.

**Conclusion:**

This QIP achieved a notable 16% reduction in huddle duration, enhancing clinical operations on the ward. The progress, combined with improved staff satisfaction and maintained quality of discussions, underscores the QIP's success in boosting clinical efficiency and offers valuable insights for future initiatives in similar settings.

